# Role of *Snord116* in pituitary growth hormone deficiency of Prader-Willi syndrome

**DOI:** 10.1172/jci.insight.205467

**Published:** 2026-09-22

**Authors:** Gabriel F. Batzli, Kaiying Guo, Fahrünisa Meryem Betül Erol, Charles A. LeDuc, Lisa C. Burnett, Rudolph L. Leibel, Yiying Zhang

**Affiliations:** 1 Department of Pediatrics, Division of Molecular Genetics, Columbia University Irving Medical Center, New York, New York, USA.; 2 Naomi Berrie Diabetes Center, Columbia University Irving Medical Center, New York, New York, USA.; 3 Foundation for Prader-Willi Research, Covina, California, USA.

**Keywords:** Development, Endocrinology, Genetics, Genetic diseases, Noncoding RNAs, Transcriptomics

## Abstract

Prader-Willi syndrome (PWS) is a complex genetic disorder resulting from the deficiency of several maternally imprinted genes, including *SNORD116*, in the 15q11-q13 region. Loss of *Snord116* in mice recapitulates some of the most salient clinical features of PWS, including growth hormone (GH) deficiency and hypogonadism. This study explored the impact of *Snord116* deficiency on early postnatal pituitary development and growth in *Snord116*-KO mice. *Snord116* was found to be expressed in both the anterior and posterior pituitary. Pituitary transcriptomes of *Snord116*-KO and WT mice at 2 developmental stages, P0 and 4 weeks of age, were interrogated and related to ex vivo analyses of GH secretion in the pituitaries of 5-week-old mice. Significant differences in pituitary transcriptomes were detected between *Snord116*-KO and WT mice at 4 weeks of age but not at P0. The differentially expressed genes and affected molecular pathways play important roles in regulating embryonic and postnatal pituitary development. Our results suggested that PWS GH deficiency was mainly due to pituitary hypoplasia and decreased GH production but not to reduced GH secretory function per se, implicating *Snord116* in the specific molecular/cellular pathways that account for impaired postnatal pituitary development and GH deficiency in PWS.

## Abstract 2

*Snord116* is a small non-coding RNA deficient in Prader-Willi Syndrome. That deficiency is implicated here in altered pituitary development, accounting for characteristic growth hormone insufficiency.

## Introduction

Prader-Willi syndrome (PWS) is a complex genetic disorder resulting from the loss of expression of a group of imprinted genes in the 15q11-q13 chromosomal region (1, 2). *SNORD116* is 1 of the 3 paternally expressed noncoding RNAs located in the 118 kb imprinted minimal critical genetic interval of PWS and is deleted or silenced in all individuals with PWS (3–6). Among their molecular roles, SNORDs are involved in 2′-*O*-methylation of small noncoding RNAs (snRNAs), such as rRNAs, by guiding 2′-*O*-methylation complexes to their RNA targets though base-pairings. SNORD-guided 2′-O-methylation is essential for the normal function and stability of these snRNAs (7–11). *Snord116* is considered an orphan because its physiological target(s) and molecular functions have not been identified (12, 13). Deletion of *SNORD116* (*Snord116*-KO) in mice recapitulates some of the most salient clinical features observed in individuals with PWS (2, 14, 15). *Snord116*-KO mice display canonical PWS traits, including postnatal growth deficiency, delayed sexual maturation, improved glucose metabolism, late-onset but mild hyperphagia, and motor-learning disability (14).

*SNORD116* is highly expressed in the hypothalamus of both mice and humans. *SNORD116* deficiency is associated with a range of behavioral, neuroendocrine, and metabolic anomalies that are attributable to impaired hypothalamic functions (16–19). *Snord116*-deficient mice display early postnatal growth retardation by P3 (14). This is remarkable because growth in neonatal rodents is highly regulated. *Ghrhr*-deficient (*lit/lit*) mice (with no positive growth regulation from the hypothalamic hormone GHRH) do not manifest reduced length growth velocity until 15 days of age (20, 21). Growth hormone deficiency (1, 22, 23), as well as decreased pituitary size and changes in pituitary morphology, have been observed in individuals with PWS (24–27). Together, these observations suggest that *SNORD116* action in extra-hypothalamic sites, such as the pituitary, may contribute to the growth deficiency in PWS.

This study explored the impact of *Snord116* deficiency on pituitary development and early postnatal growth in *Snord116*-KO mice. We found that *Snord116* was expressed in both anterior and posterior lobes of the mouse pituitary. We examined the pituitary transcriptome of *Snord116*-KO mice at 2 developmental stages, P0 and 4 weeks of age, and related these findings to ex vivo analyses of growth hormone (GH) secretion in the pituitaries of 5-week-old mice. Although no significant differences in P0 pituitary transcriptomes were found between *Snord116*-KO and WT mice, we identified significant quantitative and qualitative differences in pituitary transcriptomes between *Snord116*-KO and WT mice at 4 weeks of age. Many of the differentially expressed genes (DEGs) and affected molecular pathways play important roles in regulating embryonic and postnatal pituitary development. Our results suggest that in 4/5-week-old *Snord116*-KO mice, GH deficiency is mainly due to pituitary hypoplasia and decreased GH production but not to reduced GH secretary function per se. These results implicate impaired pituitary development in the pathogenesis of GH deficiency in PWS.

## Results

### Snord116 deficiency results in early postnatal growth retardation.

**Figure 1:**
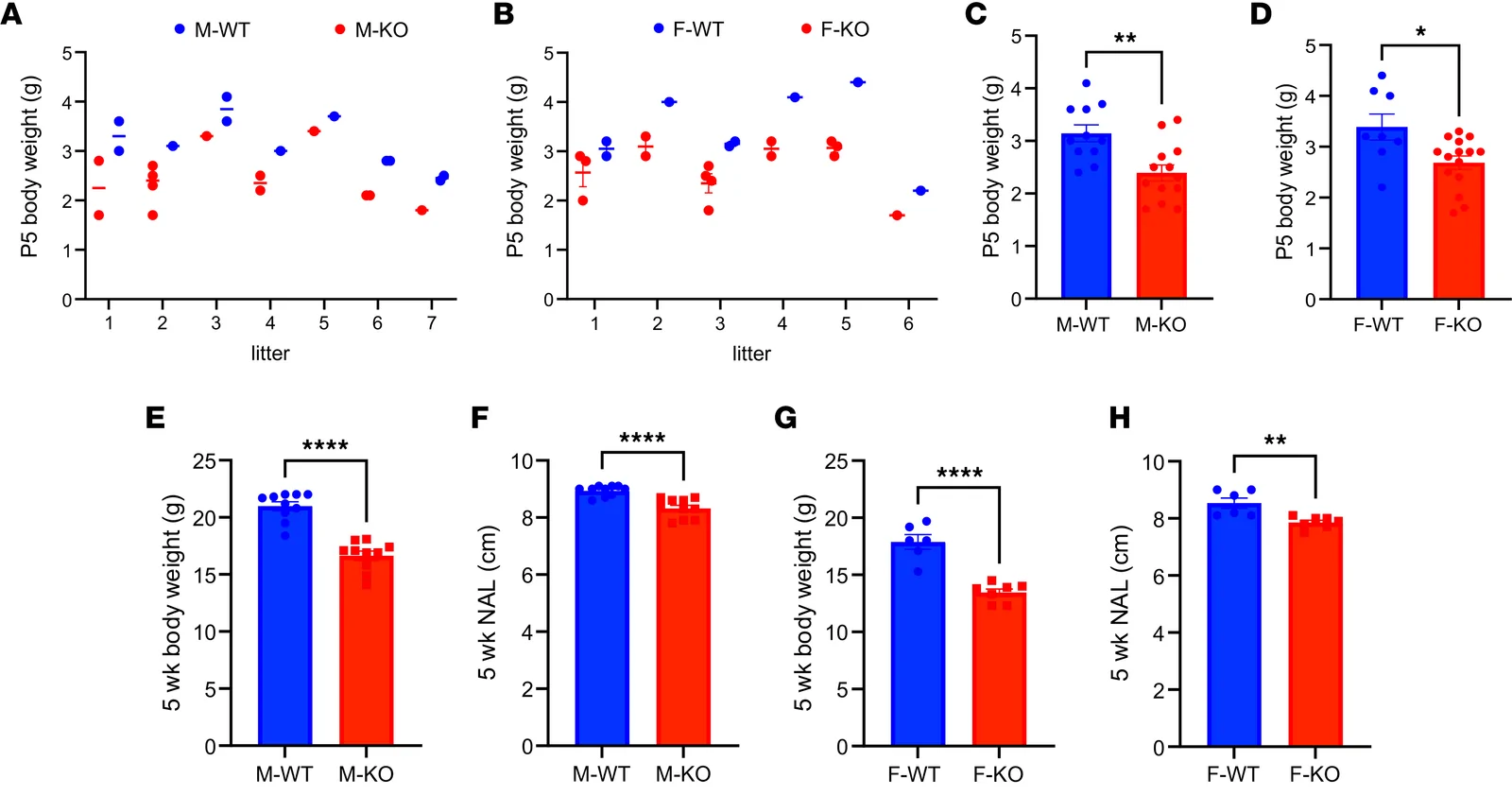
*Snord116*-KO mice present early postnatal growth retardation. (**A** and **B**) Body weight of male (**A**) and female (**B**) cohorts at P5. Genotype effect was significant in both males (*P* < 0.01) and females (*P* < 0.001) by 2-way ANOVA with genotype and litter size as grouping variables (**A** and **B**). (**C** and **D**) Body weight of male (**C**, *n* = 11–13) and female (**D**, *n* = 8–15) pups pooled from litters shown in **A** and **B**, respectively. (**E** and **F**) Body weight and naso-anal length (NAL) of 5-week-old male mice (*n* = 8). (**G** and **H**) Body weight and NAL of 5-week-old female mice (*n* = 6–7). Data are expressed as mean ± SEM; *P < 0.05, **P < 0.01, ***P < 0.001, and ****P < 0.0001; KO versus WT by 2-tailed *t* test (**C**–**H**).

The first clinical signs of PWS are failure to thrive and growth retardation in early infancy (1, 2). *Snord116*-KO mice also manifest growth retardation early in life (14). We compared body weights of pups at P5 in male and female cohorts (Figure 1, A and B). By 2-way ANOVA with genotype and litter size as grouping variables, the genotype effect was significant in both males and females (*P* < 0.01 and 0.001, respectively). In male *Snord116*-KO P5 pups, body weight was 24% lower than WT controls (2.39 ± 0.15 vs. 3.15 ± 0.16 g) (Figure 1C). Female *Snord116*-KO mice were 21% lighter than WT controls (2.69 ± 0.13 vs. 3.39 ± 0.26 g) (Figure 1D). Weighing the pituitary at this age is problematic since these pituitaries are fragile and had to be dissected in buffer to preserve integrity. As measured by the area under the dissecting microscope, the pituitary at P5 was approximately 19% smaller in *Snord116*-KO mice (Supplemental Figure 1; supplemental material available online with this article; https://doi.org/10.1172/jci.insight.205467DS1). Growth retardation of *Snord116*-KO mice persisted from P5 to 4–5 weeks of age. In a 4-week-old male cohort, body weight was 20.7% lower in *Snord116*-KO mice relative to their WT littermates (16.7 ± 0.4 vs. 21.0 ± 0.4 g, *P* < 0.0001), and body length (naso-anal length) was 6.9% lower (8.32 ± 0.11 vs. 8.94 ± 0.06 cm, *P* < 0.0001) (Figure 1, E and F). Similarly, in 5-week-old female *Snord116*-KO mice, body weight (13.44 ± 0.32 vs. 17.88 ± 0.64, *P* < 0.0001) and naso-anal length (7.86 ± 0.08 vs. 8.53 ± 0.18, *P* < 0.01) were 25% and 8% lower relative to WT controls (Figure 1, G and H).

### Snord116 is expressed in both anterior and posterior pituitary.

**Figure 2:**
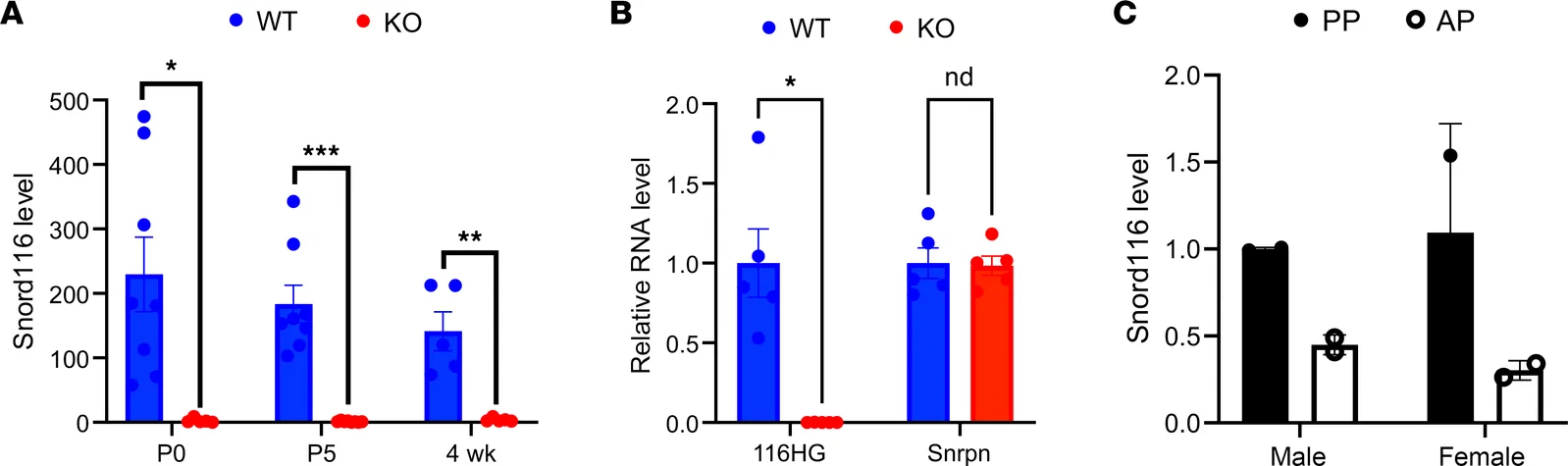
*Snord116* is expressed in the mouse pituitary throughout life and in both posterior and anterior pituitary. (**A**) *Snord116* transcript levels (AU) in the WT and *Snord116*-KO mouse pituitary at P0 (*n* = 5–8), P5 (*n* = 8), and 4 weeks of age (*n* = 5). (**B**) Relative 116HG RNA levels and *Snrpn* mRNA levels in the pituitaries of 4-week-old male mice. The average WT level is defined as 1. Data are expressed as mean ± SEM; *P < 0.05, **P < 0.01, ***P < 0.001; KO versus WT by 2-tailed *t* test (**A** and **B**). (**C**) *Snord116* levels normalized to housekeeping gene *Ppia* in the posterior pituitary (PP) and anterior pituitary (AP) of 12-week-old WT mice as determined by qRT-PCR.

The neuroendocrine dysfunction observed in individuals with PWS, such as growth retardation and hypogonadism, has been considered to be primarily hypothalamic in origin (1, 2). However, the early postnatal growth retardation and pituitary hypoplasia observed in *Snord116*-KO mice resemble the growth retardation phenotype of *Snell* and *Ames* dwarf mice, which are caused by defects in the key transcription factors mediating pituitary growth: *Pou1f1* (*Pit1*) and *Prop1* (28, 29). In contrast, growth retardation in *little* mice, which is due to a mutation in the cognate receptor for GHRH (*Ghrhr*), is not detected until P15 (20, 21). Furthermore, *Nkx2.1*Cre-mediated *Snord116* deletion, which causes pan-hypothalamic *Snord116* KO, including in the tuberal region of the hypothalamus where GHRH-secreting neurons are located, causes milder growth retardation compared with the germline *Snord116* deletion used here. Thus, it is likely that intrinsic *Snord116* action in the developing pituitary causes hypoplasia, accounting for the early postnatal growth retardation phenotype. Although S*NORD*116 expression has been detected in many extra-hypothalamic sites in humans (30), it has been unclear whether *Snord116* is expressed in the pituitary of either mice or humans and directly regulates postnatal pituitary development. We found that *Snord116* transcript levels were markedly higher in the WT than in *Snord116*-KO pituitaries (virtually undetectable) at P0, P5, and 4 weeks of age (Figure 2A). No significant age-related differences in *Snord116* transcript level were observed between P0 and 4 weeks of age in WT mice by 1-way ANOVA (Figure 2A). As expected, *Snord116* KO also led to markedly decreased transcript levels of 116HG in the pituitary, the spliced product of the *Snord116* host gene (Figure 2B). mRNA levels of *Snrpn*, located upstream of the *Snord116* cluster and outside of the minimal critical PWS interval but derived from the same primary transcript as *Snord116* and *116HG* (3–6), were not significantly different between the WT and *Snord116*-KO pituitaries (Figure 2B).

*Snord116* transcript levels were assessed in freshly isolated anterior and posterior pituitaries from 12-week-old WT mice. *Snord116* transcripts were detected in the reverse transcription (RT) samples of both anterior and posterior pituitaries, and *Snord116* PCR signals were barely detectable in the respective RT-controls, in which the reverse transcriptase was omitted (Supplemental Figure 2), indicating that *Snord116* detection in the RT samples was not an artifact of genomic DNA contamination. *Snord116* levels in the anterior pituitary, normalized to the housekeeping gene *Ppia*, were about 50% of those of the posterior pituitary in both males and females (Figure 2C).

### Snord116 deficiency is associated with an altered balance between cell proliferation and differentiation that is reflected in the transcriptome of immature pituitaries.

**Figure 3:**
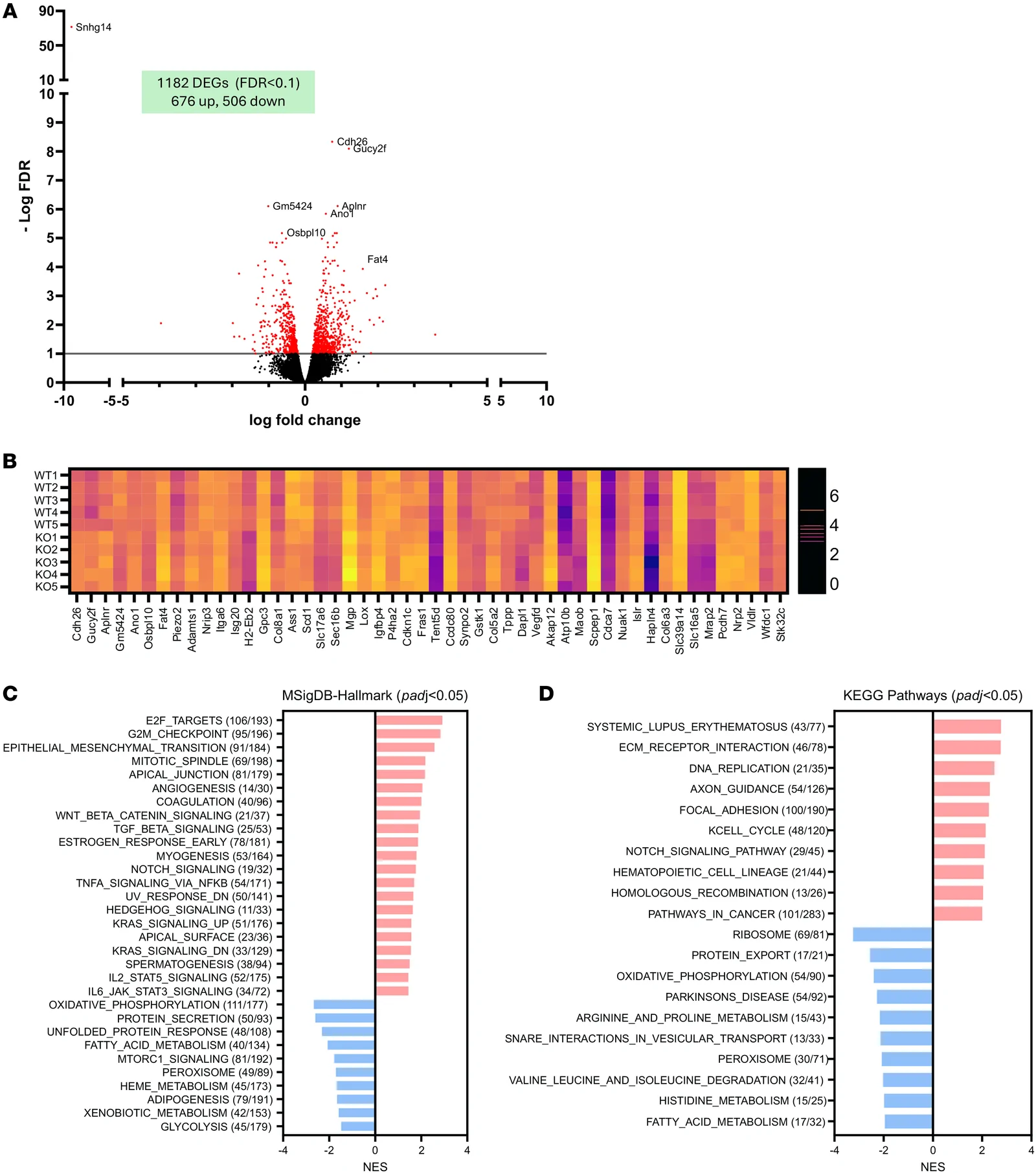
Transcriptomic analysis of pituitaries from 4-week-old male *Snord116*-KO mice and WT controls. (**A**) Volcano plot of DEGs. The total number of DEGs (FDR < 0.1) and numbers of upregulated and downregulated DEGs in *Snord116*-KO pituitaries relative to WT controls are shown in the insert. (**B**) Heatmap of the top 50 most significant DEGs (FDR < 0.0005, excluding the *Snord116* host gene, *Sngh14*) between *Snord116*-KO and WT pituitaries, 22 downregulated and 28 upregulated. The color bars show log_2_-transformed counts per million values of the transcripts. (**C**) Significantly enriched MSigDB-Hallmark pathways/processes in *Snord116*-KO pituitaries relative to WT controls (adjusted *P* < 0.05). (**D**) Top 10 positively and negatively enriched KEGG pathways/processes in *Snord116*-KO pituitaries relative to WT controls (adjusted *P* < 0.05). The number of leading-edge genes relative to the size of the gene set for each enriched pathway is in parentheses following the name of the pathway in **C** and **D**. Light red bars represent positively enriched pathways. Light blue bars represent negatively enriched pathways. NES, normalized enrichment score.

We performed transcriptomic profiling of *Snord116*-KO and WT pituitaries by RNA-Seq at P0 and 4 weeks of age. No significant differences in the transcriptomes of *Snord116*-KO and WT pituitaries were detected in P0 pups other than the transcript encoded by the deleted *Snord116* locus, that is, *Snord116* host gene *Snhg14* (log_2_ fold-change [FC] = –6.9, FDR < 0.0001). Quantitative RT-PCR analysis of the P0 pituitary RNA samples used for the RNA-Seq study confirmed that levels of 3 transcripts from the *Snord116* locus, *Snord116*, *116HG*, and *IPW*, were markedly decreased in *Snord116*-KO relative to WT mice, whereas *Snrpn* mRNA levels were not significantly different between the 2 groups (Supplemental Figure 3). In pituitaries of 4-week-old mice, 1,182 DEGs (FDR < 0.1) were detected: 676 upregulated and 506 downregulated in *Snord116*-KO mice relative to WT controls (Figure 3A) (Supplemental Table 1). A heatmap of the top 50 DEGs is shown in Figure 3B. *H19* (log_2_ FC = 2.2, FDR < 0.001) and *Igf2* (log_2_ FC = 1.3, FDR < 0.015), 2 important regulators of early postnatal organ development, were among the most upregulated (relative to WT) DEGs in the *Snord116*-KO pituitary (Supplemental Table 1). *Snhg14* (log_2_ FC = –9.15, FDR < 0.0001), *Gpr101* (log_2_ FC = –1.25, FDR < 0.005), and *Lhb* (log_2_ FC = –1.165, FDR = 0.027) were among the most downregulated DEGs in *Snord116*-KO pituitaries (Supplemental Table 1). *Gpr101* is a developmentally regulated gene that has been implicated in the regulation of tonic GH secretion by mature somatotrophs (31–33). *Lhb*, the gene encoding the β-subunit of luteinizing hormone, is a marker gene for gonadotrophs. *Gh* transcript level (log_2_ FC = –0.56, FDR = 0.26) decreased in *Snord116*-KO pituitaries relative to WT controls, but the difference did not reach statistical significance. Many of the DEGs have been implicated in pituitary development, such as *Prop1* (log_2_ FC = 0.57, FDR = 0.055) and *Pou1f1* (also known as *Pit1*) (log_2_ FC = –0.27, FDR = 0.078), 2 key developmental pituitary transcription factors for which hypomorphic alleles result in dwarfism (34) (Supplemental Table 1).

Gene set enrichment analysis (GSEA) of 4-week-old pituitary transcriptomes indicated that 21 and 10 Molecular Signatures Database (MSigDB) Hallmark pathways/processes were, respectively, positively and negatively enriched in *Snord116*-KO relative to WT mice (adjusted *P* < 0.05). The top positively enriched MSigDB Hallmark processes (increased in KO) included gene sets involved in the regulation of proliferation and development, such as E2f targets, G2M checkpoint, epithelial-mesenchymal transition, mitotic spindle, and genes responding to K-RAS signaling, as well as signaling pathways known to regulate these processes, such as Wnt/β-catenin, TGF-β, Notch, and hedgehog signaling (Figure 3C). Positively enriched Hallmark gene sets in *Snord116*-KO pituitaries also included genes encoding the components of apical junction and surface as well as genes involved in angiogenesis, myogenesis, and hormone/cytokine signaling (Figure 3C). The top negatively enriched (decreased in KO) processes were involved in metabolism. These included pathways for oxidative phosphorylation and fatty acid metabolism. Protein secretion, unfolded protein response, and mTORC1 signaling, which plays an important role in regulating protein synthesis, were also among the negatively enriched processes in *Snord116*-KO pituitaries (Figure 3C). Twenty-five Kyoto Encyclopedia of Genes and Genomes (KEGG) pathways were positively enriched and 25 were negatively enriched (adjusted *P* < 0.05) (Supplemental Table 2); top 10 hits of each set are shown in Figure 3D. Many of the enriched KEGG processes/pathways overlapped with the enriched Hallmark processes (Figure 3, C and D). Additionally, KEGG pathways/processes involved in cell adhesion and migration, such as axon guidance, focal adhesion, and regulation of actin cytoskeleton, were positively enriched in *Snord116*-KO pituitaries (Figure 3D). The negatively enriched KEGG processes included genes involved in protein export and amino acid metabolism as well as those encoding components of the cellular organelles involved in fatty acid and protein metabolism, such as peroxisome, lysosome, and proteosome (Figure 3D). The GSEA results suggest that pituitaries from 4-week-old *Snord116*-KO mice were more active in cell proliferation and early developmental programs than those of the WT controls and were less active in substrate and energy metabolism, suggesting that *Snord116*-KO pituitaries may be less mature than their WT counterparts.

**Figure 4:**
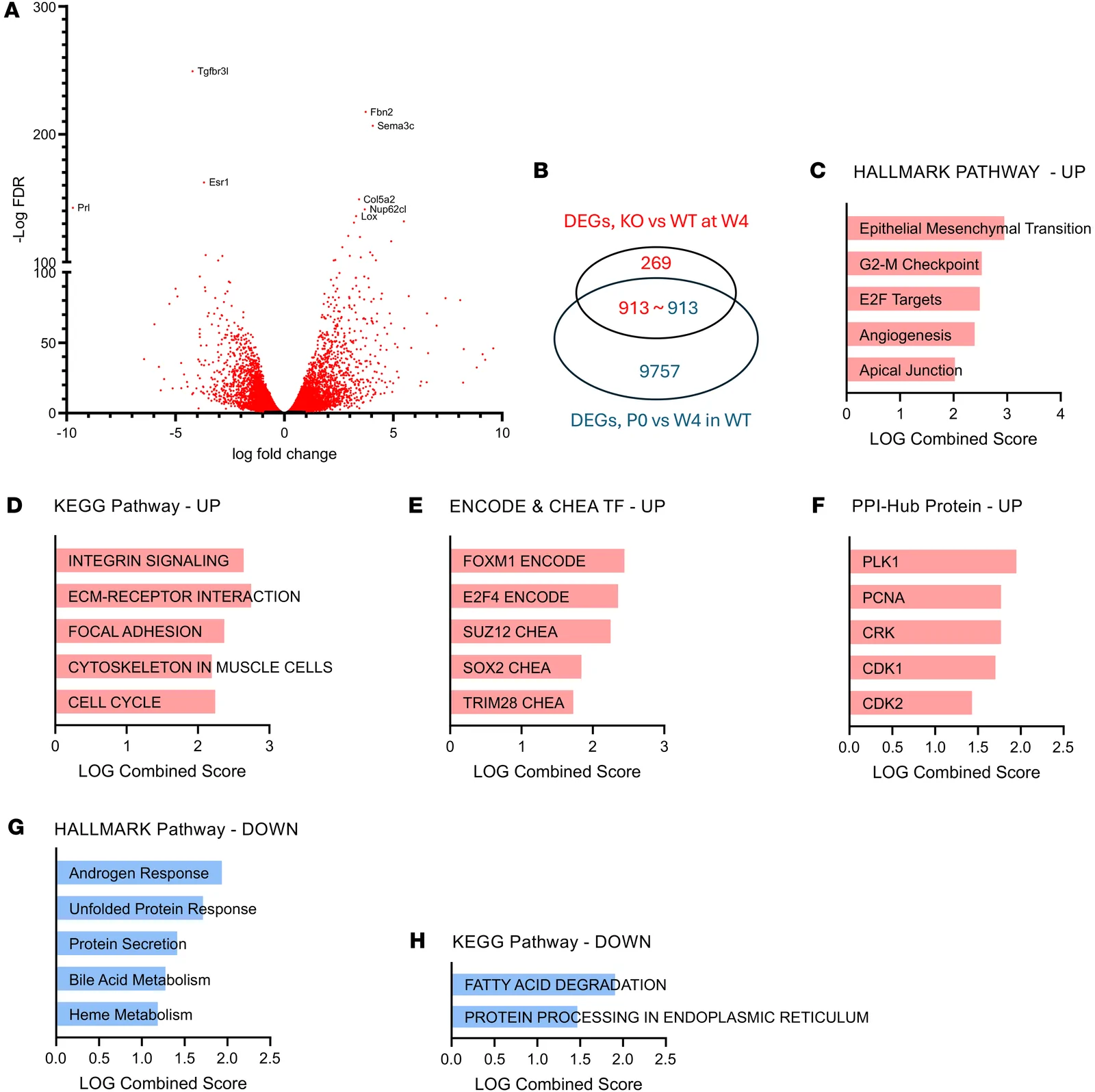
Transcriptomic features shared by male 4-week-old *Snord116*-KO and immature P0 WT pituitaries. (**A**) Volcano plot of a total of 10,670 DEGs from P0 versus 4-week-old (W4) WT pituitaries (FDR < 0.1). (**B**) 913 DEGs were shared in the same directions among a total of 1,182 DEGs from *Snord116*-KO (KO) versus WT at 4 weeks of age (W4) and a total of 10,607 DEGs from P0 versus W4 in WT. Among the shared 913 DEGs, 541 were upregulated and 372 were downregulated in both P0_WT and W4_KO relative to W4 _WT. (**C**–**F**) Enrichr analysis results of the 541 upregulated DEGs shared by P0_WT and W4_KO relative to W4_WT (adjusted *P* < 0.05) (light red bar graphs), enriched HALLMARK pathways (**C**), enriched KEGG pathways (**D**), enriched ENCODE and ChIP enrichment analysis (ChEA) consensus transcription factors (TFs) from ChIP-X (**E**), and enriched protein-protein interaction (PPI) hub proteins (**F**). (**G** and **H**) Enrichr analysis results of the 372 downregulated DEGs shared by P0_WT and W4_KO relative to W4_WT (adjusted *P* < 0.05) (light blue bar graphs), enriched HALLMARK pathways (**G**), and enriched KEGG pathways (**H**). No significant enriched ENCODE and ChEA consensus TFs from ChIP-X or PPI hub proteins were detected by Enrichr among the shared 372 downregulated DEGs. The combined scores were calculated by Enrichr based on Fisher’s exact test *P* value and *z* score for deviation from the expected rank, reflecting the significance of the enrichment. The combined scores were then log_10_-transformed and plotted.

To identify transcriptomic changes associated with normal postnatal pituitary development and maturation, we compared the transcriptomes of P0 and 4-week-old pituitaries from WT mice. A total of 10,670 DEGs were identified (FDR < 0.1) (Figure 4A, Supplemental Table 3). These DEGs, as a signature gene set of “developing and immature pituitary,” were compared with the DEGs from 4-week-old *Snord116*-KO versus WT mice, as a signature gene set of “*Snord116*-deficiency in 4-week-old pituitaries.” In P0 WT pituitaries and 4-week-old *Snord116*-KO pituitaries versus 4-week-old WT pituitaries, 913 DEGs from the 2 signature gene sets overlapped; that is, they were changed in the same direction (541 upregulated and 372 downregulated) (Figure 4B) (Supplemental Table 4), representing 77% of DEGs between 4-week-old *Snord116*-KO versus WT mice. Fisher’s exact test indicated that the percentage of overlapping DEGs in *Snord116*-KO versus WT mice at 4 weeks of age (913 out of 1,182 DEGs, 77.2%) was significantly higher than expected by chance when compared with the number of DEGs (10,670 DEGs out of 16,045 genes examined, 66.5%) between P0 and 4-week-old in WT mice (*P* < 0.0001, OR = 1.71, 95% CI: 1.49–1.96) (Supplemental Table 5). Key pituitary transcription factors, such as *Prop1* and *Pou1f1*; components of the Notch signaling pathway, such as *Notch1*, *Notch3*, *Jag1*, and *Dll1*; and regulators of early postnatal growth, such as *H19* and *Igf2*, were all among the shared DEGs (Supplemental Table 4). *Prop1* expression decreased (log_2_ FC = 2.33, FDR < 0.0001, P0 vs. week 4 [W4] in WT), and *Pou1f1* increased (log_2_ FC = –0.55, FDR < 0.0001, P0 vs. W4 in WT) during normal postnatal pituitary development from P0 to 4 weeks of age in WT mice. Enrichr analyses showed that pathways related to the regulation of cell cycle and developmental programing, such as epithelial-mesenchymal transition and G2-M checkpoint, predominated in the upregulated DEGs shared by P0 and 4-week-old *Snord116*-KO pituitaries (Figure 4, C and D), while protein secretion, unfolded protein response, and fatty acid degradation pathways predominated in the shared downregulated DEGs (Figure 4, G and H). Consistent with the enrichment of MSigDB Hallmark pathways/processes that regulate cell proliferation, targets of transcription factors *Foxm1*, *Suz12*, and *E2F4*, which are activators of cell proliferation/division, were enriched in the shared upregulated DEGs (Figure 4E), and so were several protein-protein interaction (PPI) hub proteins, such as *PLK1*, *PCNA*, and *CDK1* (Figure 4F), which have been implicated in regulating cell division, DNA replication, and cell cycle progression. Taken together, these results suggest that *Snord116* deficiency in the pituitary alters the balance between cell proliferation and differentiation during early postnatal pituitary development, leading to delay and/or attenuation of terminal differentiation/maturation of the hormone-secreting pituitary cells, including somatotrophs in the anterior pituitary.

**Figure 5:**
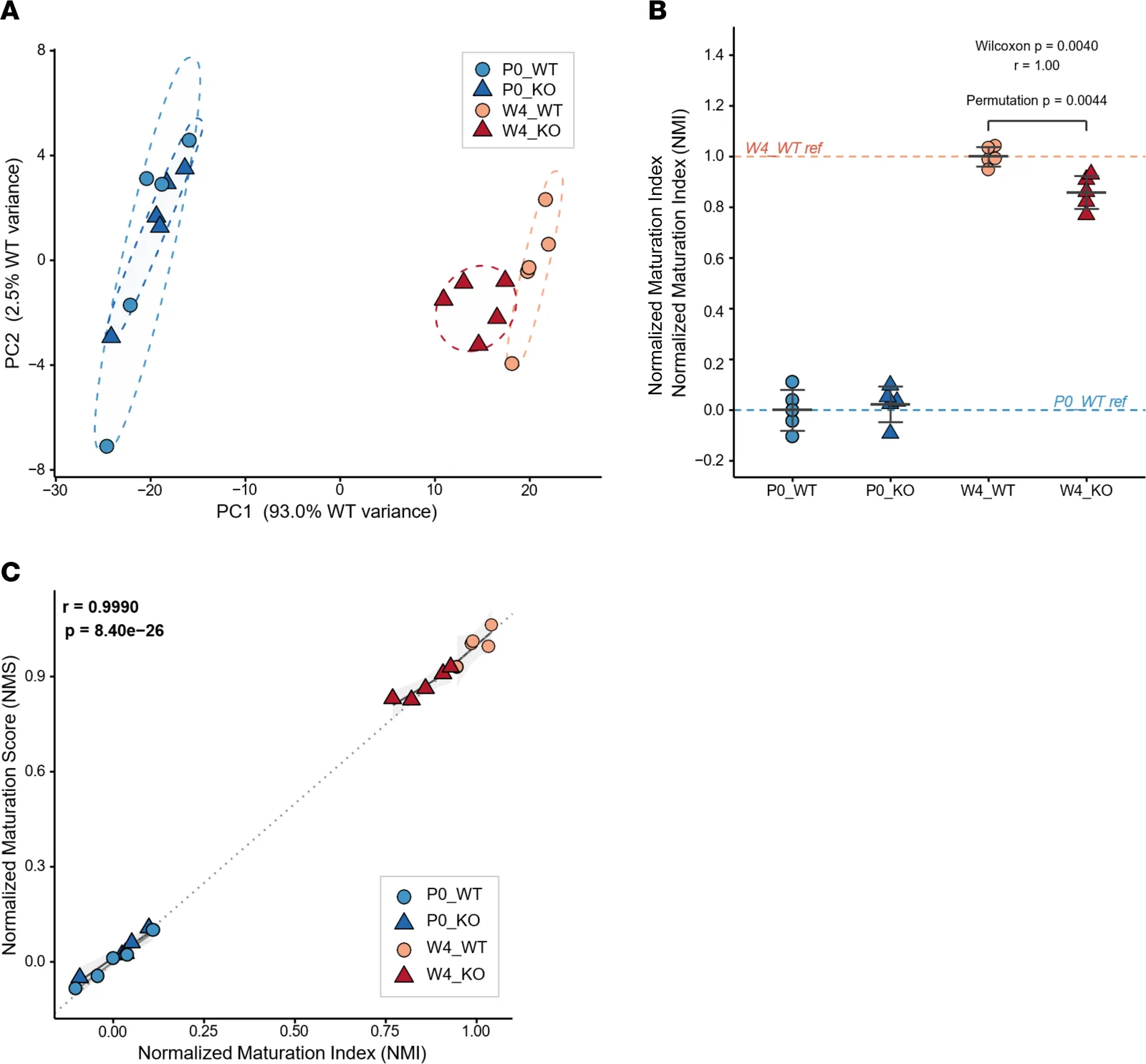
Transcriptomic analysis shows molecular signature of attenuated postnatal pituitary maturation in *Snord116*-KO mice. (**A**) WT-reference PCA of pituitary RNA-Seq data from WT and *Snord116*-KO mice at P0 and 4 weeks of age (W4). The PCA space was defined using WT samples, and KO samples were then projected into this space. PC1 (93.0% variance) represents the postnatal maturation axis. (**B**) PCA-based NMI, which was scaled to the mean P0_WT and W4_WT reference states. W4_KO pituitaries reached only 86% of the W4_WT maturation level, which was statistically significant by 1-sided Wilcoxon’s rank-sum test and permutation test (both *P* < 0.01). (**C**) Concordance between the PCA-based NMI and the PCA-independent NMS of all samples, *r* = 0.9990, 95% CI: 0.9974–0.9996, *P* = 8.40 × 10^–26^, by Pearson’s correlation analysis.

To quantitatively analyze the maturity of 4-week-old *Snord116*-KO (W4_KO) pituitaries relative to WT controls at P0 and 4 weeks of age (P0_WT and W4_WT), we first examined global transcriptomic relationships across all samples using standard principal component analysis (PCA) with the top 500 highly variable genes. PC1 explained 85.1% of the variance and separated P0 from W4 pituitaries with strong overlap between P0_WT and P0_KO samples and a detectable shift of W4_KO samples relative to W4_WT controls (Supplemental Figure 4). To quantify the position of W4_KO samples along the normal WT pituitary maturation trajectory, we constructed a WT reference PCA framework to capture the normal WT developmental trajectory without influence from KO-specific transcriptional variation. PCA was trained exclusively on WT samples using the top 500 highly variable genes between P0_WT and W4_WT; KO samples were then passively projected into this WT-defined coordinate system (Figure 5A). PC1, which explained 93.0% of the variance between P0_WT and W4_WT pituitaries, was interpreted as the principal postnatal maturation axis in WT. Again, P0_KO pituitaries largely overlapped with P0_WT samples along this axis, whereas W4_KO samples shifted away from W4_WT samples and occupied a relatively less mature position along the WT-defined maturation axis (Figure 5A). Normalized maturation index (NMI) calculated by rescaling each sample’s WT-reference PC1 score relative to the P0_WT and W4_WT group means, which were defined as 0 and 1, respectively, indicated that W4_KO pituitaries reached a mean NMI of 0.858 ± 0.065, compared with 1.000 ± 0.039 in W4_WT controls (Figure 5B). This difference was statistically significant by a 1-sided Wilcoxon’s rank-sum test (W = 25, *P* = 0.0040; rank-biserial *r* = 1.00, indicating complete rank separation) and a permutation test (*P* = 0.0044). We further validated the NMI result using a PCA-independent normalized maturation score (NMS), which was calculated based on WT maturation-associated genes (i.e., DEGs between P0_WT and W4_WT). W4_KO pituitaries reached a mean NMS of 0.870 ± 0.047 (Supplemental Figure 5), again indicating incomplete maturation relative to W4_WT controls. NMI and NMS were almost perfectly correlated across all 20 samples (Pearson’s *r* = 0.9990, 95% CI: 0.9974–0.9996, *P* = 8.40 × 10–26) (Figure 5C), demonstrating that the PCA-based maturation index (NMI) and the independent gene-signature score (NMS) captured the same biological signal.

### Decreased basal and GHRH-stimulated GH secretions in Snord116-KO pituitaries are due to decreased pituitary size and GH production.

**Figure 6:**
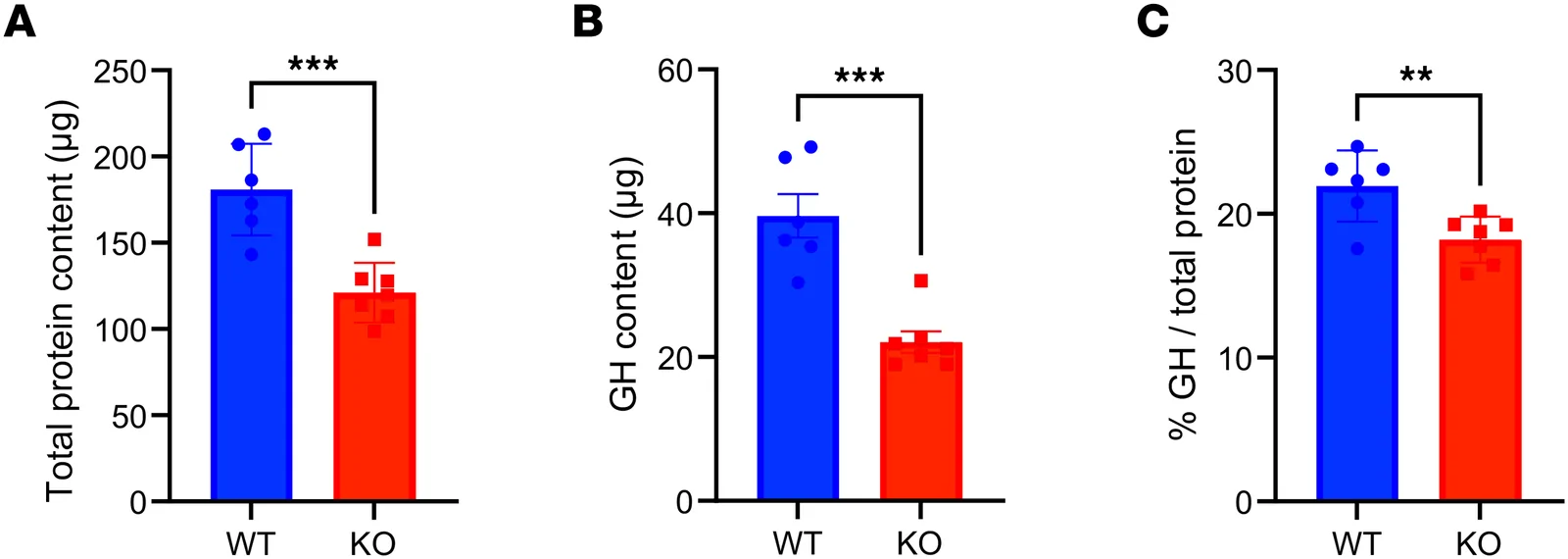
Pituitary total protein and GH are decreased in 5-week-old female *Snord116*-KO mice relative to WT controls. (**A**) Pituitary total protein content. (**B**) Pituitary GH content. (**C**) GH content normalized to total protein content (*n* = 6–7). Data are expressed as mean ± SEM; **P < 0.01 and ***P 0.001; KO versus WT by 2-tailed *t* test.

**Figure 7:**
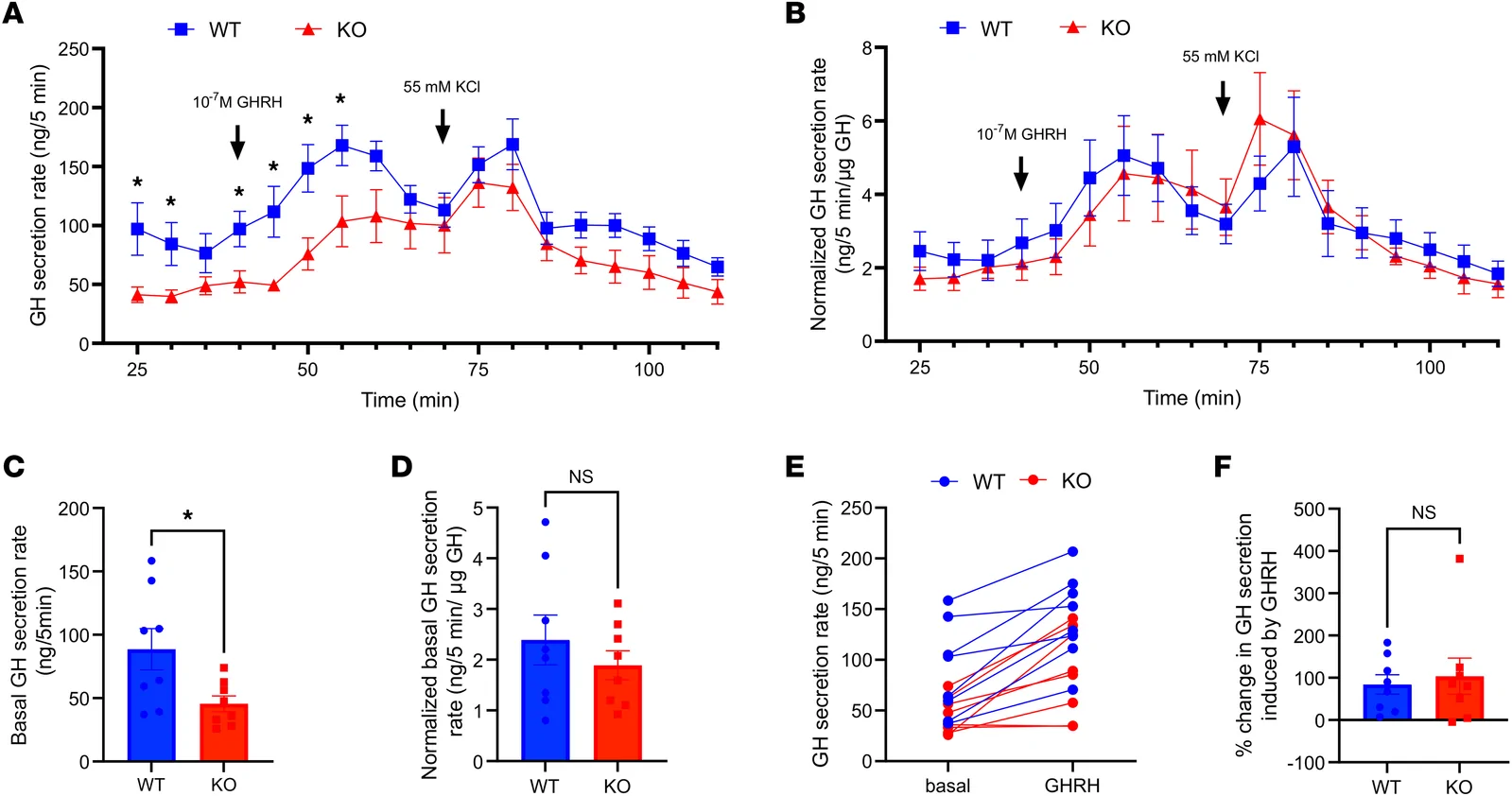
GH secretion in the basal and GHRH-stimulated states in pituitaries of 5-week-old male *Snord116*-KO and WT mice. (**A**) GH secretion rate (ng/5 min) during the perifusion experiments (*n* = 8). The time point when a 5-minute perfusion with medium containing 10^–7^ M GHRH or 55 mM KCl is indicated. (**B**) GH secretion rate in **A** was normalized to the tissue GH content. (**C**) Basal GH secretion rate was averaged from effluent collected between the 25- and 40-minute time points. (**D**) The average basal GH secretion rate after normalization to tissue GH content. (**E**) The change in the average GH secretion rates in the basal state (25–40 minutes) and after GHRH stimulation (45–70 minutes) in the *Snord116*-KO and WT pituitaries. (**F**) The average fold increases in GH secretion rate after GHRH stimulation. Data are expressed as mean ± SEM; *P < 0.05; KO versus WT by 2-tailed *t* test.

We investigated GH protein production and secretion in *Snord116*-KO and WT pituitaries. In 5-week-old female *Snord116*-KO pituitaries, total protein and GH content were, respectively, 33% (*P* < 0.001) and 44.4% (*P* < 0.001) lower than those of WT pituitaries (Figure 6, A and B). GH content normalized to total protein content was 27% (*P* < 0.01) lower in the mutant pituitaries relative to WT (Figure 6C). Similar results were also observed in the pituitary extracts from 5-week-old male mice after they had been subjected to perifusion (Supplemental Figure 6). These results indicated that *Snord116*-KO mice had decreased pituitary size with lower GH content relative to pituitary cell mass. In 5-week-old male mice, absolute amounts of GH secreted during the perifusion of isolated pituitaries in the basal state and after a bolus stimulation with 10^–7^ M GHRH were significantly lower in the *Snord116*-KO versus WT mice (Figure 7A). When GH secretion rates were normalized to pituitary GH content (Supplemental Figure 6A), there was no significant difference between *Snord116*-KO and WT pituitaries in either the basal or GHRH-stimulated state (Figure 7B). The mean absolute GH secretion rate in the basal state (between 25 and 40 minutes) was significantly lower (*P* < 0.01) in the KO mice (Figure 7C); this difference was eliminated by normalizing with GH content (Figure 7D). GHRH increased GH secretion by 84% (WT) and 104% (*Snord116*-KO) relative to the basal state, but the difference was not statistically significant (*P* = 0.69; Figure 7, E and F). In the aggregate, these results suggest that decreased pituitary cell mass and maturity, not the GH secretory function of the cells, is the primary cause of GH deficiency in *Snord116*-KO mice.

## Discussion

### Summary of main findings/conclusion.

*Snord116* deficiency in mice results in pituitary hypoplasia that is accompanied by postnatal GH-deficient growth retardation. In 4- to 5-week-old mice, we found that pituitary GH content relative to pituitary size was disproportionally decreased in *Snord116*-KO mice: a 33% reduction in pituitary protein content (a surrogate for pituitary size) and a 44.3% reduction in GH content. These quantitative differences suggest that pituitary hypoplasia in the mutant mice is likely associated with a reduced number of GH-producing cells at this developmental stage. Ex vivo GH secretion analysis in 5-week-old male mice indicated that GH deficiency in *Snord116*-KO mice is primarily due to pituitary hypoplasia and decreased GH synthesis; GH secretory function per se was minimally affected either in the basal state or in response to GHRH stimulation in *Snord116*-KO mice. It is reasonable to assume that GH secretion results obtained in the prepubertal male pituitaries are extrapolatable to the females given that all other growth and pituitary phenotypes of the PWS mouse model examined in this study were not sexually dimorphic. Growth deficiency is not a sexually dimorphic trait in individuals with PWS either (1, 2).

**Figure 8:**
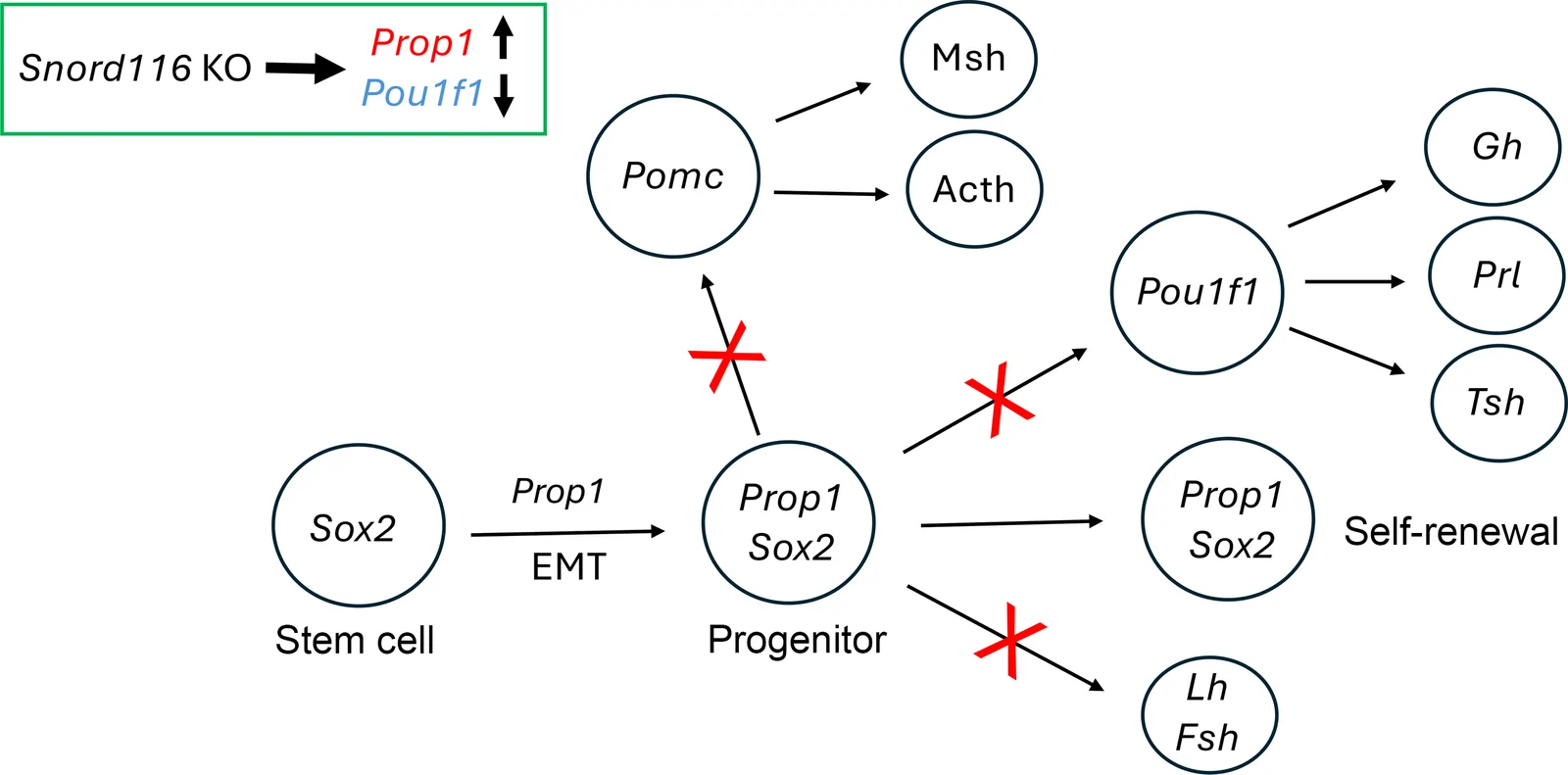
*Snord116* may influence the development of hormone-producing cells in the anterior pituitary by regulating *Prop1* expression/function. *Prop1* is a pituitary-specific paired-like homeodomain transcription factor. The expression of *Prop1* activates epithelial-mesenchymal transition during the early stages of pituitary development that is essential for the development of all hormone-producing cells in the anterior pituitary; downregulation of *Prop1* expression in the later developmental stages is required for the terminal differentiation of gonadotrophs (50, 51). *Prop1* expression decreases during normal postnatal pituitary development from P0 to 4 weeks of age in WT mice. *Snord116* deficiency leads to elevated *Prop1* expression and decreased expression of *Pou1f1*, a transcription factor that is activated by *Prop1* and is required for the terminal differentiation of somatotrophs, lactotrophs, and thyrotropes, in 4-week-old *Snord116*-KO pituitaries relative to WT controls (box in upper left). Although it is possible that the changes in *Prop1* and *Pou1f1* expression in 4-week-old *Snord116*-KO pituitaries may reflect the decreased maturity of KO pituitaries, we hypothesize that *Snord116* may regulate *Prop1* expression/function in *Prop1*-expressing progenitor cells, and that the elevated (or dysregulated) *Prop1* expression in these cells due to *Snord116* deficiency delays and/or attenuates the terminal differentiation of all hormone-producing cells, resulting in attenuated postnatal pituitary expansion and maturation.

Contrary with our results, an earlier study found no significant differences in the number of somatotrophs and GH immunogenicity between *Snord116*-KO and WT mice at 4 weeks of age (14). The conflicting results may reflect, in part, methodological differences: the respective abilities of ELISA and IHC staining to accurately quantify pituitary GH content. In our study, GH concentration in the tissue lysate was measured by ELISA, and tissue GH content was calculated by multiplying lysate GH concentration with the volume of tissue lysate, which accounted for changes in both pituitary size and GH production rate. In contrast, IHC staining used in the previous study could not accurately quantify changes in GH production rate or pituitary size (14). Buttressing our inference, human pituitary size (measured by MRI) is about 50% smaller in children and young adults with PWS compared with unaffected individuals of the same age (27). Our profiling of pituitary gene expression also suggests that differentiation of gonadotrophs was affected in *Snord116*-KO mice, consistent with the delayed vaginal opening in female mutant mice (14), suggesting that *Snord116* deficiency may affect the cellular ontogeny of anterior pituitary hormone–producing cells at or prior to the divergence of terminal differentiation of gonadotrophs and cells of *Pou1f1* lineage (somatotrophs, lactotrophs, and thyrotropes) during the early postnatal period (35) (Figure 8).

We have demonstrated that *Snord116* is expressed in both anterior and posterior lobes of the pituitary throughout life, albeit at much lower levels than those found in the brain based on the Ct values of qRT-PCR with similar RNA inputs from the 2 tissues (unpublished observations). Low levels of *Snord116* expression in pituitaries of 5-week-old mice suggest that *Snord116* expression may be limited to a subpopulation of pituitary cells. At 4 weeks of age, *Snord116* deficiency is associated with gene expression profiles that are remarkably similar to those of pituitaries from WT newborn pups: high in proliferative progenitor cells and low in hormone-producing cells. PCAs of pituitary transcriptomes confirmed that *Snord116*-KO pituitaries are less mature than WT controls at 4 weeks of age. These results suggest that *Snord116* deficiency attenuates the postnatal differentiation/expansion of hormone-producing cells in the anterior lobe, contributing to growth retardation and other neuroendocrine dysfunctions of *Snord116*-KO mice.

### Characteristics of postnatal growth retardation in Snord116-KO mice.

Body weights of *Snord116*-KO mice are not different from WT littermates at birth; pituitary size and morphology appear normal at E18.5 (14). We found that pituitary size and gene expression profiles were not significantly different between *Snord116*-KO and WT mice at birth, indicating that pituitary hypoplasia and growth retardation in *Snord116*-KO mice begin after birth. The postnatal growth retardation phenotype observed in *Snord116*-KO mice resembles that of the *Ames* (*df*) and *Snell* (*dw*) dwarfs in that growth retardation manifests during the early postnatal period and is accompanied by pituitary hypoplasia (36–38). *Ames* dwarf and *Snell* dwarf mice segregate for recessive mutations in the *Prop1* and *Pou1f1* (aka *Pit1*) genes, respectively, which encode 2 key pituitary-specific transcription factors required for the expansion and differentiation of hormone-producing cells in the anterior lobe (28, 29, 35). Both *Prop1* and *Pou1f1* transcript levels were also altered, increased and decreased, respectively, in *Snord116*-KO pituitaries relative to WT controls. Like *Snord116*-KO mice, *Snell* dwarf mice do not differ in body weight or length from WT mice at birth but quickly develop severe growth retardation due to virtually complete absence of somatotrophs and GH (28). In contrast, growth retardation in mice segregating for a missense mutation in *Ghrhr*, which encodes the anterior pituitary somatotroph receptor for the hypothalamic GHRH, becomes detectable later, around P15 (20, 21, 39). These differences suggest that in *Snord116*-KO mice, the early postnatal growth retardation is unlikely to be related to impaired hypothalamic function. Consistent with this inference, the growth retardation phenotype is significantly milder in mice with *Nkx2.1* Cre-mediated conditional hypothalamic deletion of *Snord116* (unpublished observations). Adult body weights of *Snord116*-KO mice are approximately 85% of WT mice (14), whereas the adult body weight of mice with *Nkx2.1* Cre-mediated conditional deletion of *Snord116* is about 92% of WT mice. These differences suggest that *Snord116* deficiency in extra-hypothalamic sites contributes to the postnatal growth retardation in *Snord116*-KO mice.

### Mechanistic insights on the role of Snord116 action in postnatal pituitary development from transcriptomic profiling.

Mouse pituitary mass expands dramatically during the first 3 postnatal weeks through proliferation and differentiation of pituitary progenitor cells (40–42). Although pituitary transcriptomes of *Snord116* P0 pups were not significantly different from WT (outside the targeted manipulation), by 4 weeks of age these transcriptomes were markedly different from each other. The most upregulated pathways in 4-week-old *Snord116*-KO pituitaries were those related to the regulation of cell cycle progression and epithelial-mesenchymal transition, processes essential for activating and mobilizing pituitary stem/progenitor cells prior to their terminal differentiation into hormone-producing cells (43, 44). The components of several signaling pathways, such as hedgehog, *Wnt*/β catenin, and Notch, which regulate embryonic and early postnatal pituitary development (45), were also upregulated in pituitaries from 4-week-old *Snord116*-KO mice, presumably reflecting delayed/attenuated pituitary development and maturation in *Snord116*-KO mice.

The pituitary transcriptomes of 4-week-old *Snord116*-KO mice resemble those of P0 WT (or *Snord116*-KO) pituitaries. Furthermore, 77% of the DEGs between *Snord116*-KO and WT pituitaries at 4 weeks of age were also differentially expressed in the same direction in WT P0 pituitaries when compared with 4-week-old WT pituitaries. Gene ontology analysis of the shared upregulated DEGs again points to the enrichment of genes involved in regulating cell cycle progression and epithelial-mesenchymal transition, underscoring similarities between 4-week-old *Snord116*-KO pituitaries and the immature P0 WT pituitaries. *Sox2*^+^ pituitary stem cells are highly proliferative during the first 2–3 weeks of life in concordance with major organ growth (46–49). *Prop1*, the pituitary-specific and paired-like homeodomain transcription factor mutated in *Ames* dwarf mice (29), stimulates *Sox2*^+^ stem cells to undergo epithelial-mesenchymal–like transition, cell migration, and proliferation prior to their differentiation toward hormone-producing cells (43). *Prop1* also plays an important role in regulating *Sox2* expression and stem cell proliferation critical for the maintenance of self-renewal of pituitary stem cells (43). We found that *Prop1* transcript, which was decreased during normal postnatal pituitary development in the WT mice from P0 to 4 weeks of age, was upregulated in *Snord116*-KO pituitaries relative to WT controls at 4 weeks. Temporal control of *Prop1* expression is critical for the development of gonadotrophs (50, 51). In humans, mutations in *Prop1* are associated with combined pituitary hormone deficiency that includes TSH, PRL, GH, GN, and ACTH deficiency (52–56), resembling the endocrine abnormalities observed in PWS (1, 2). We found that *Pou1f1*, which acts downstream of *Prop1* and is critical for the terminal differentiation of somatotrophs, lactotrophs, and thyrotropes, was downregulated along with decreased expression of *Lhb*, a marker of gonadotrophs, in *Snord116*-KO mice. These observations, along with the upregulated expression of cell cycle and epithelial-mesenchymal transition genes, suggest that *Snord116* may regulate *Prop1* expression and/or function. As shown in Figure 8, although acknowledging that the exact relationship between *Snord116* deficiency and elevated *Prop1* expression is unknown, we speculate that elevated and/or dysregulated *Prop1* expression in *Snord116*-KO pituitary may attenuate or delay the terminal differentiation of all hormone-producing cells in the anterior pituitary, leading to multiple neuroendocrine abnormalities in *Snord116*-KO mice and in individuals with PWS.

Genes involved in oxidative phosphorylation, fatty acid oxidation, and glycolysis, as well as protein secretion and unfolded protein response, were downregulated in 4-week-old *Snord116*-KO compared with WT pituitaries. Consistently, genes encoding the components of cellular organelles specialized in performing these processes, such as peroxisome, proteosome, and lysosome, were also downregulated. These specific transcriptomic profiles likely reflect the relative immaturity of *Snord116*-KO pituitaries and decreased numbers of hormone-producing cells, which are specialized in the energy-demanding cellular processes of producing and secreting hormones.

In contrast with the results obtained in pituitaries of 4-week-old *Snord116*-KO mice, in the P5 hypothalamus of *Snord116*-KO mice, genes involved in protein synthesis and secretion were upregulated, whereas those involved in regulating cell cycle progression were downregulated (unpublished observations). These results suggest that the effects of *Snord116* are tissue type– and developmental stage–specific and that an assumption of universality may lead to false inferences from negative experiments.

*Gpr101* is a highly downregulated gene in 4-week-old *Snord116*-KO pituitaries. *Gpr101* induces GH secretion via Gs and Gq/11 signaling pathways involving both PKA and PKC (32). Duplication of *Gpr101* causes X-linked acrogigantism in humans; transgenic overexpression of the gene in somatotrophs causes hypersecretion of GH and prolactin and gigantism in mice (31, 32, 57). In rats, *Gpr101* is co-expressed with GH in somatotrophs (58). In the Rhesus monkey and mouse, *Gpr101* is expressed mainly in *Gnrhr*-expressing cells, that is, gonadotrophs (Single cell portal — Broad Institute) (59). In *Snord116*-KO mice, decreased *Gpr101* expression, together with decreased expression of hormone-specific luteinizing hormone β subunit (*Lhb*), suggest that gonadotroph differentiation is affected by *Snord116* deficiency. Consistent with the pituitary gene expression profile, vaginal opening was delayed by 3.6 days in *Snord116*-KO mice (14), recapitulating the gonadal axis neuroendocrine abnormalities found in patients with PWS (1, 2). The combined deficiencies in gonadotrophs and somatotrophs in *Snord116*-KO pituitaries strengthen the notion that *Snord116* acts at a point in the postnatal pituitary differentiation process before the divergence of these 2 specific hormone-producing cells, as shown in Figure 8 (35).

In summary, *Snord116* acts in the anterior pituitary during the early postnatal period to modulate postnatal expansion and maturation of hormone-producing cells. The growth retardation phenotype and delayed sexual maturation in *Snord116*-KO mice, and transcriptomic profiles of P0 and P4 pituitaries, suggest that *Snord116* may influence postnatal pituitary expansion and differentiation by modulating *Prop1* expression and/or cellular processes regulated by *Prop1*, such as self-renewal of Sox2^+^ progenitor cells, the epithelial-mesenchymal transition, and consequently, terminal differentiation of hormone-producing cells in the anterior pituitary (Figure 8). It is possible that instances of panhypopituitarism in humans might be due to developmental somatic inactivation of *SNORD116*.

The posterior pituitary, which also expresses *Snord116*, plays an important role in regulating the secretion of neuropeptide oxytocin. Oxytocin deficiency is a canonical PWS phenotype. The potential role of *Snord116* in regulating the development/function of the posterior pituitary and the oxytocin axis warrants further study.

The pituitary cell population(s) in which *Snord116* is expressed is currently unknown. Available pituitary single-cell sequencing datasets have no information on *Snord116* expression because the current single-cell RNA-Seq technology is unable to co-profile small RNAs with mRNAs. However, new strategies are being developed to simultaneously co-profile small RNA and mRNA at single-cell resolution (60, 61). After the cell populations in which *Snord116* is expressed are identified, single-cell RNA-Seq could assess cell-autonomous effects of *Snord116* KO in *Snord116*-expressing cells and paracrine effects of *Snord116* KO in distinct populations of non–*Snord116*-expressing cells across different developmental stages, providing potentially new insights into molecular mechanisms by which *Snord116* regulates the development of different pituitary cell populations.

### Limitations of this study.

The anatomic, molecular, and ex vivo perifusion data here clearly implicate *Snord116* in ontogeny of pituitary somatotrophs. We did not, however, measure the basal and stimulated GH secretion in vivo in WT and *Snord116* hypomorphic animals at P5 or week 4. Early postnatal malnutrition may cause subsequent growth retardation. As we and others have shown, the severe early onset postnatal growth retardation in *Snord116*-KO mice — characterized by a 25% reduction in body weight associated with a 44% reduction in pituitary GH content at 5 weeks of age — persists from P5 to 4 weeks of age. However, there is significant catch up in growth after weaning, eventuating in only a moderate 15% reduction of body weight in adult *Snord116*-KO mice. Infants with PWS display hypotonia and feeding/swallowing difficulty resulting in early failure to thrive (1, 2). It is possible that *Snord116*-KO mice have similar feeding difficulties during the early postnatal period, contributing to the growth retardation phenotype that is more severe during the early postnatal period than in adulthood. Ding et al. reported no significant difference in the size of the gastric milk spot between *Snord116*-KO and WT pups, and the mutant mice appeared to have no hypotonia when tested at P5 and 3 weeks of age (14). Thus, additional factors, such as malabsorption, may also contribute to the growth retardation phenotype of *Snord116*-KO mice. Infants with PWS also have diarrhea and other gastrointestinal symptoms (1, 2). Based on our observation that the number of ghrelin-producing gastric cells is increased in *Snord116*-KO mice (unpublished observations), it is possible that *Snord116* deletion may affect the differentiation of gastrointestinal stem cells, impairing both neuroendocrine and absorptive functions. Additionally, we cannot rule out the possibility that *Snord116* deletion may affect the expression of other noncoding RNAs in the vicinity, contributing to the pituitary phenotype observed in the current study.

## Methods

### Sex as a biological variable.

Both male and female mice were included in the study. The specific PWS phenotypes, including postnatal growth retardation, pituitary hypoplasia, and decreased pituitary GH content, were observed in both sexes of the PWS mouse model used in this study, consistent with the absence of sexual dimorphism in growth deficiency in individuals with PWS. Pituitary RNA-Seq was performed using RNA samples from male mice at P0 and 4 weeks of age. Ex vivo GH secretion analysis (perifusion assay) was performed in 5-week-old male pituitaries. The age-matched female pituitaries were freshly isolated and flash-frozen to obtain pituitary GH contents of the KO mice and their WT controls without subjecting them to perifusion assay to avoid potential artifacts associated with such ex vivo experimentation. Given that the growth phenotype examined in this study is not a sexually dimorphic trait, we assumed that the results obtained from one sex are extrapolatable to the other sex. The conclusions of this study are relevant to both sexes.

### Reagents and chemicals.

The total RNA Purification Plus Micro kit was from Norgen Biotek (catalog 48500). iQ SYBR Green Supermix was from Bio-Rad. M-MLV reverse transcriptase, random hexamer, Pierce BCA Protein Assay kit, and all tissue culture reagents were obtained from Thermo Fisher Scientific. DNA oligos for RT-PCR and TaqMan probes were from Integrated DNA Technology. GHRH (18-053-PS) was purchased from Peptide Sciences. Mouse Growth Hormone ELISA kit (EZRMGH) was purchased from MilliporeSigma. All other chemicals were from Sigma-Aldrich.

### Animal husbandry, genotyping, and tissue collection.

*B6.Cg-Snord116^tm1.1Uta^/J* mice (stock 008149, *Snord116* deletion line) were obtained from The Jackson Laboratory. Male heterozygous *B6.Cg-Snord116^tm1.1Uta^/J* mice were crossed with female WT C57BL/6J mice to generate *Snord116*-KO mice (*Snord116^p-/m^*) and WT littermates for molecular and phenotype characterizations. Ear notch or tail DNAs were used for genotyping as previously described (14). A compound TaqMan assay for *Sry*, a gene located on the Y chromosome (present only in males), and for a chromosome 6 DNA segment (present in both sexes), was used to determine the sex of young pups. The sequences of primers and probes used for genotyping and sex determination are listed in Supplemental Table 6. All mice were kept in a barrier facility and maintained at 22°C with a 12-hour light/12-hour dark cycle with ad libitum access to rodent breeder chow (Picolab, 5058) and water. Mice were euthanized with CO_2_ asphyxia followed by cervical dislocation. After euthanasia, blood was collected by cardiac puncture using EDTA as an anticoagulant, and pituitaries were collected, flash-frozen in liquid nitrogen, and stored at –80°C for molecular analyses or used immediately for ex vivo GH secretion assays.

### RNA-Seq and data analysis.

Total RNA was extracted using Total RNA Purification Plus Micro kit following the manufacturer’s instructions. RNA quality was assessed using an Agilent Bioanalyzer 2100. Only samples with RNA integrity numbers greater than 9 were used for RNA-Seq. Ribo-Zero Plus rRNA Depletion Kit and TruSeq Stranded Total RNA Library Prep Kit Gold (Illumina) were used for library preparation before cDNA was sequenced on a NovaSeq 6000 with paired-end 100 bp read length and 40 million read counts per sample. Library preparation and RNA-Seq were performed by JP Sulzberger Columbia Genome Center. RNA-Seq data were processed using Galaxy Version 21.01 (62). Data quality was assessed using FastQC, and reads with quality scores less than 20 were removed from the dataset. Reads were aligned to the Mouse GRCm39 genome using HISAT2, and aligned reads were counted over genomic features using FeatureCounts (63, 64). Differential expression analysis was performed using EdgeR with minimal counts per million of 0.5 in the number of samples equal to or greater than that of the smaller sample number (*n*) of the 2 groups being compared (65). The statistical significance threshold was adjusted *P* value (FDR) less than 0.1 using Benjamini-Hochberg algorithm (66). GSEA was performed using Galaxy fGSEA tool and curated MSigDB Hallmark and KEGG gene sets (67, 68).

For standard PCA, WT-reference PCA, and maturation-index/maturation-score analyses, count matrices were first transformed using the variance-stabilizing transformation (VST) implemented in DESeq2. VST was performed with blind = FALSE to account for the experimental design. Standard PCA was performed on all 20 samples using the top 500 highly variable genes, selected by row-wise variance across the blind = FALSE VST matrix. PCA was performed using the prcomp function with centering enabled and scaling disabled. WT-reference PCA was performed using the top 500 highly variable genes based on row-wise variance calculated across WT samples only. Expression values for these genes were then mean-centered and scaled using WT-specific gene means and standard deviations. PCA was performed on the WT-standardized WT expression matrix using prcomp with no additional centering or scaling. This approach defines the maturation axis using only WT samples, thereby preventing KO-specific transcriptional variation from influencing the reference coordinate system. As a result of WT-reference PCA, PC1 explained 93.0% of the WT variance and was interpreted as the WT pituitary maturation axis. All WT and KO samples were then passively projected into this WT-defined PCA space using the WT-derived rotation matrix without reestimating the principal components.

NMI was defined as NMI*_i_* = (PC1*_i_* – meanPC1_P0_WT_)/(meanPC1_W4_WT_ – meanPC1_P0_WT_), where PC1*_i_* is the WT-reference PC1 projection score for sample *i*. This normalization sets the mean P0_WT score to 0 and the mean W4_WT score to 1, allowing W4_KO samples to be interpreted as their relative fractional position along the normal WT postnatal pituitary maturation axis. The difference in NMI between W4_WT (*n* = 5) and W4_KO (*n* = 5) was evaluated using 2 complementary nonparametric tests. First, a 1-sided Wilcoxon’s rank-sum test was performed to test the hypothesis that W4_WT samples have higher NMI values than W4_KO samples. The test was conducted using R’s Wilcox.test function with exact = TRUE. Effect size was reported as the rank-biserial correlation, calculated as *r* = 2U ⁄ (n_1_n_2_) −1, where *U* is the Mann-Whitney *U* statistic. Second, a permutation test with 10,000 label permutations was used to estimate the probability of observing a W4_WT minus W4_KO mean difference equal to or greater than the observed value under the null hypothesis of no group difference.

NMS was used as a PCA-independent validation of the NMI by calculating a transcriptomic maturation signature score from DEGs identified in the W4_WT versus P0_WT contrast (FDR < 0.05). Using the blind = FALSE VST matrix, expression values for each gene were standardized across all 20 samples to generate gene-level *z* scores. For each sample, the raw NMS was calculated as the mean *z* score of genes upregulated during WT maturation minus the mean *z* score of genes downregulated during WT maturation. The raw score was then linearly normalized so that the P0_WT group mean was set to 0 and the W4_WT group mean was set to 1, placing the NMS on the same scale as the NMI. Statistical testing was performed as described for the NMI. Agreement between the PCA-based NMI and the PCA-independent NMS was assessed across all 20 samples using Pearson’s correlation. The 95% CI for the correlation coefficient was calculated using Fisher’s *r* to *z* transformation.

### Enrichr analysis.

GSEA for DEGs was analyzed using the web-based Enrichr program (http://amp.pharm.mssm.edu/Enrichr) (69).

### qRT-PCR.

Total RNA was reverse-transcribed into first-strand cDNA using M-MLV reverse transcriptase and random hexamer. qPCR was carried out using gene-specific primers and Bio-Rad iQ SYBR Green Supermix or gene specific TaqMan assays. Two housekeeping genes, Cyclophilin A (*Ppia*) and TATA-box binding protein (*Tbp*), were used to normalize the RNA input. ΔCt was calculated using the geometric mean of *Tbp* and *Ppia* for each sample. The ΔΔCt method was used to calculate relative expression level. qPCR primers are listed in Supplemental Table 6.

### Ex vivo GH secretion assay.

Fresh pituitaries were dissected from 5-week-old male mice and incubated in DMEM/Ham F12 media supplemented with 0.1% BSA. The medium was oxygenated by bubbling carbogen (95% O_2_/5% CO_2_) through the liquid for 30 minutes before BSA addition (incubation medium). Perifusion of the pituitary was carried using a Acusyst-S Perifusion System at a flow rate of 0.125 mL/min as previously described (70). After a 20-minute preincubation with the incubation medium (effluents from 0–20 minutes were discarded), aliquots of 0.625 mL effluent were collected every 5 minutes for 20 minutes (effluents collected at 25–40 minutes were treated as the basal GH release). Pituitaries were then transiently perifused for 5 minutes with GHRH-supplemented incubation medium (10^–7^ M GHRH) and then reverted to the unsupplemented incubation medium for another 25 minutes, with effluents being collected every 5 minutes (0.625 mL) (effluents collected between 45 and 70 minutes were treated as GHRH-stimulated GH release). At the70-minute time point, pituitaries were transiently perifused for 5 minutes with KCl-supplemented incubation medium (55 mM KCl) and then switched back to the unsupplemented incubation medium for an additional 40 minutes. Effluents collected between 75 and 85 minutes were treated as KCl-stimulated GH secretion. At the end of the perifusion (110-minute time point), pituitaries were recovered, flash-frozen in liquid nitrogen, and stored at –80°C. Pituitaries from 5-week-old female mice were dissected, flash-frozen, and stored at –80°C. Pituitaries from both cohorts were then used for GH and protein content analyses.

### Pituitary protein extraction and ELISA of mouse GH.

Frozen individual pituitaries were homogenized in NP-40 lysis buffer (50 mM Tris-HCl, pH 8.0, 1% NP-40, 1 mM EDTA, 150 mM NaCl_2_, 50 mM NaF, 0.5 mM NaVO_4_, 5 mM Na_4_P_2_O_7_, and 1 mM DTT and protease inhibitors). Tissue homogenates were sonicated for 2 minutes in an ice water bath followed by centrifugation at 20,000*g* for 15 minutes to remove insoluble cellular debris. The cleared cell lysates were then used to determine GH and protein concentrations using the Mouse Growth Hormone ELISA kit from MilliporeSigma and Pierce BCA Protein Assay kit. Individual pituitary GH content and protein content were calculated by multiplying the volume of lysate with GH and protein concentrations in the lysate, respectively.

### Statistics.

RNA-Seq data were analyzed using Galaxy or R as described in *RNA-Seq and data analysis*. GraphPad Prism was used for statistical analysis of all other data. Data are shown as mean ± SEM of the indicated animal cohorts. Differences between *Snord116*-KO and WT controls were examined using 2-tailed Student’s *t* test or 2-way ANOVA, and *P* less than 0.05 was considered statistically significant.

### Study approval.

All animal procedures were in compliance with the accepted standards of animal care and were approved by the Columbia University IACUC.

### Data availability.

All the underlying data for the bar graphs are reported in the Supporting Data Values file. RNA-Seq data from pituitaries of P0 and 4-week-old *Snord116*-KO and WT mice have been deposited in NCBI’s Gene Expression Omnibus (GEO GSE333730).

## Author contributions

YZ initiated and supervised the study. GFB, RLL, and YZ designed the study. GFB, KG, FMBE, and YZ conducted experiments and acquired and analyzed data. RLL and YZ procured the funding, GFB, KG, FMBE, CAL, LCB, RLL, and YZ wrote and/or reviewed the manuscript.

## Conflict of interest

The authors have declared that no conflict of interest exists.

## Funding support

This work is the result of NIH funding, in whole or in part, and is subject to the NIH Public Access Policy. Through acceptance of this federal funding, the NIH has been given a right to make the work publicly available in PubMed Central.

This project is supported in part by grants from the following sources:

- Foundation for Prader-Willi Research (to YZ).
- Russell Berrie Foundation (to RLL).
- New York Nutrition & Obesity Research Center (P30 DK26687, to RLL).
- Columbia University Diabetes Research Center (P30 DK63608, D. Accili, PI).

## Supplementary Material

Supplemental data

Supplemental table 1

Supplemental table 2

Supplemental table 3

Supplemental table 4

Supplemental table 5

Supporting data values

## Version 1 09/22/2026

Electronic publication
