# Supplementary material for: Role of Snord116 in pituitary growth hormone deficiency of Prader-Willi syndrome
Source: JCI Insight. 2026 Sep 22;11(18):e205467. doi: 10.1172/jci.insight.205467 (PMC13596709; doi:10.1172/jci.insight.205467)
Supplement: Supplementary file 1 [file jciinsight-11-205467-s276.pdf]

Supplementary figures and materials

<u>Supplemental figures</u>

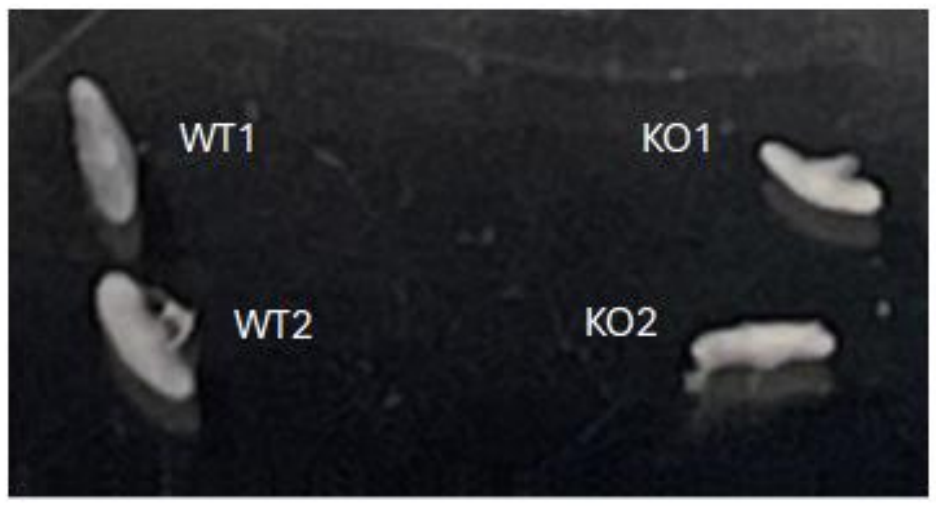

Pit ID	Area (pixel)
WT1	2362
WT2	2830
KO1	1976
KO2	2231

Fig S1. ***Snord116* KO pituitaries are smaller than those of the wild type at postnatal day 5.** A. The image of P5 pituitaries from two *Snord116* KO (KO1 and KO2) and two wild type (WT1 and WT2) pups photographed side by side. B. Quantification of the areas (in pixel) occupied by the pituitaries in the photo using ImageJ.

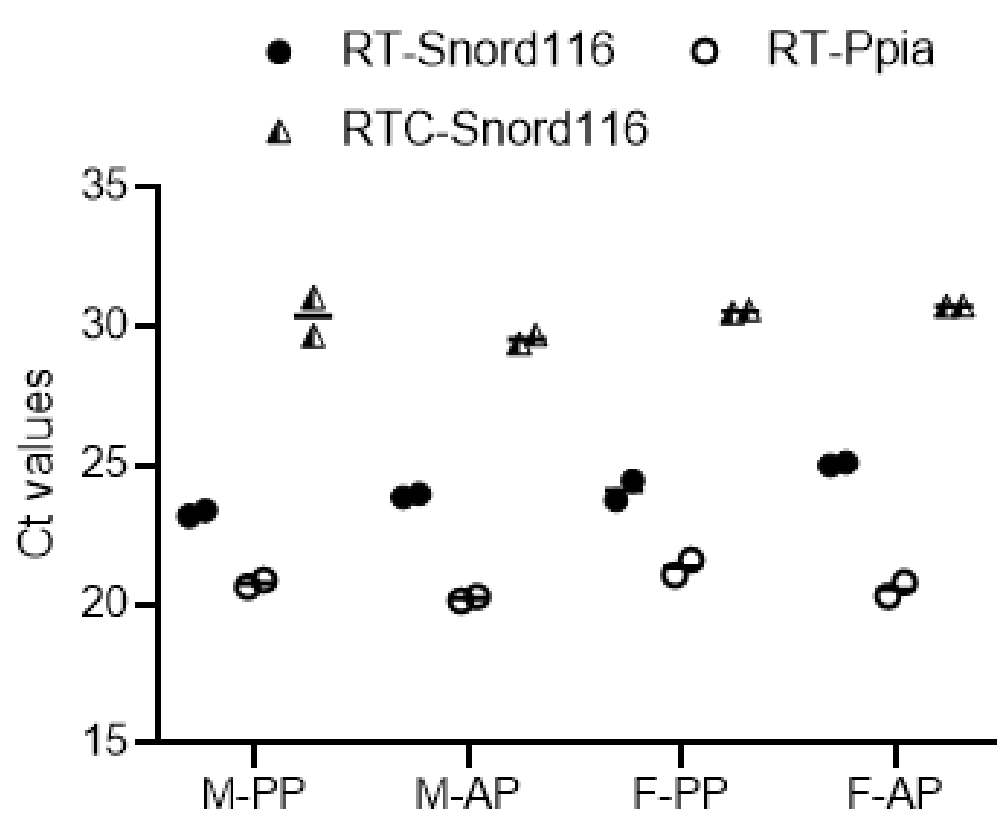

Fig S2. **Detection of *Snord116* transcripts in the anterior (AP) and posterior (PP) pituitaries**. Raw Ct values of *Snord116* and *Ppia* in the reverse transcription samples (RT) and/or the reverse transcription control samples (RTC), in which the reverse transcriptase was omitted, of the anterior (AP) and posterior (PP) pituitaries from 12-week-old wild type mice (n=2).

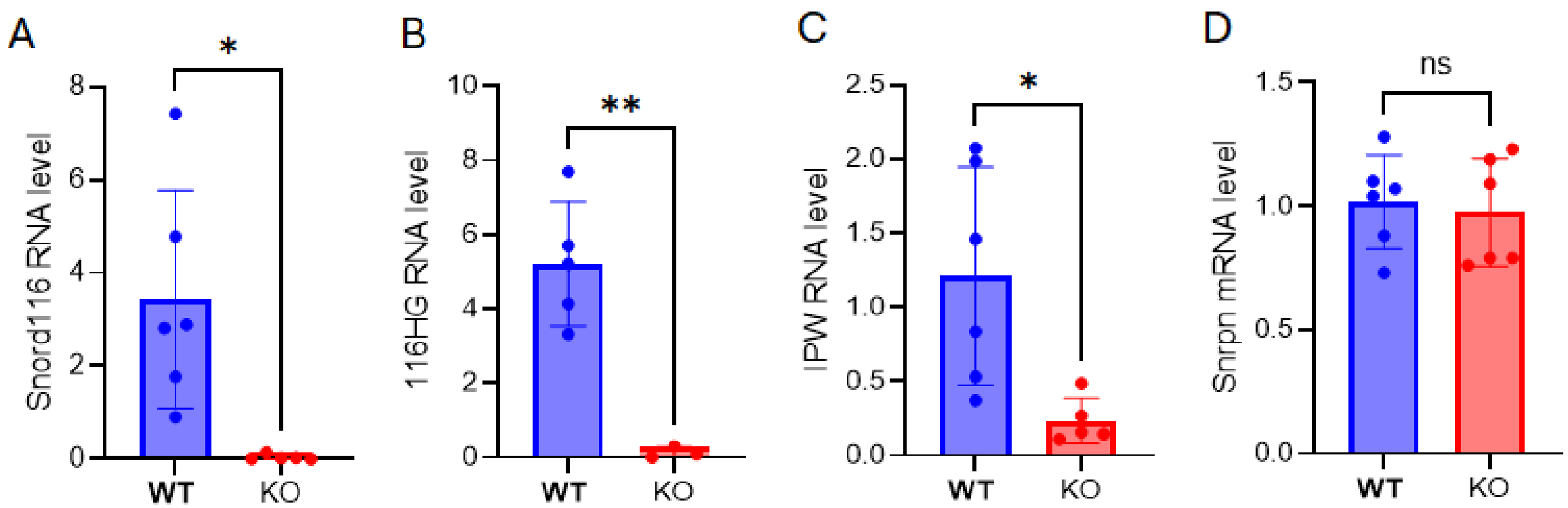

Fig S3. **qRT-PCR results of P0 pituitary RNA samples from *Snord116* KO and WT pups**. **A**. *Snord116* RNA levels (a.u.), **B**. *116HG* RNA levels (a.u.), **C**. *IPW* RNA levels (a.u.), **D**. *Snrpn* mRNA levels (a.u.). Data are expressed as Mean ± SEM (n=5-6). * and ** denote p<0.05 and 0.01, KO vs WT by two-tailed t-test.

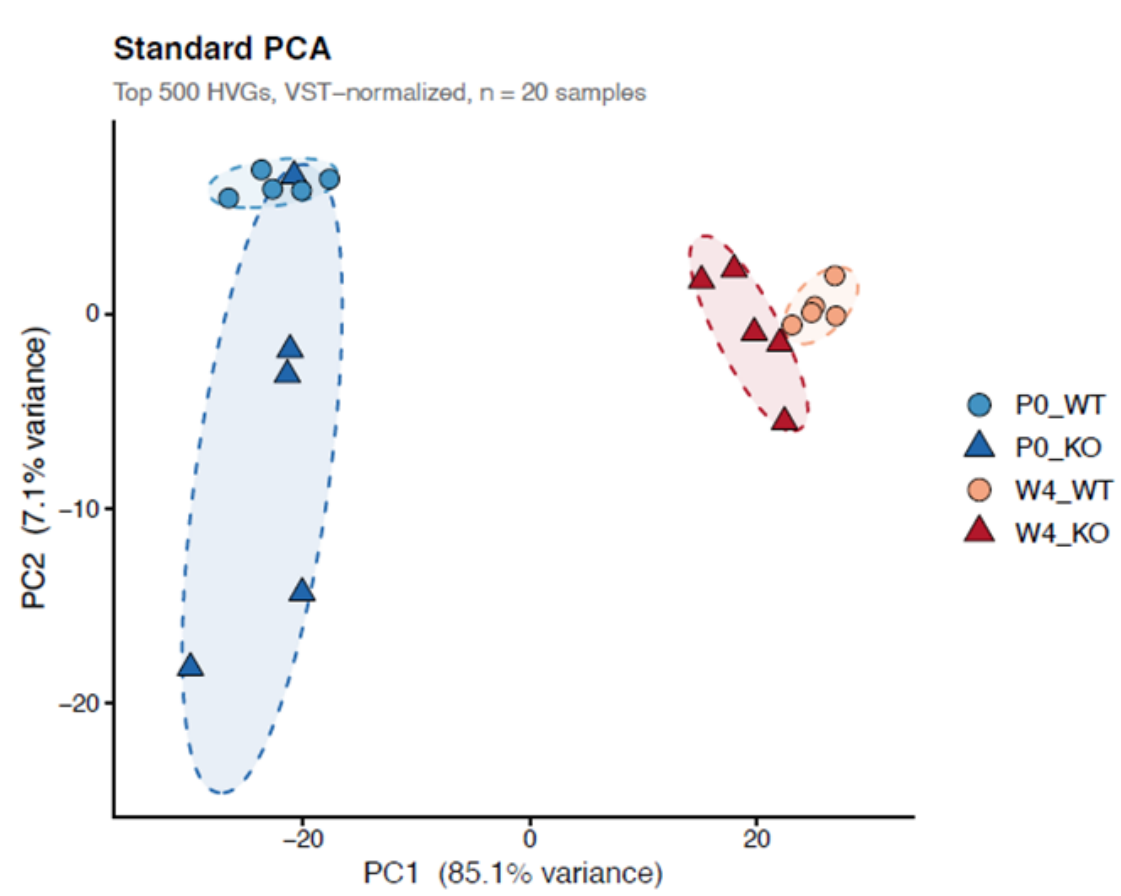

Fig S4. **Standard principal component analysis (PCA) of pituitary transcriptomes from P0 and 4-week-old *Snord116* KO mice and wild type controls.** PCA was performed across all four groups of samples, P0 and 4-week-old (W4) *Snord116* KO (KO) mice and wild type (WT) controls, using the top 500 most variable genes from VST-normalized counts. PC1 explains 85.1% and PC2 explains 7.1% of total variance. Circles denote WT and triangles denote KO samples. Separation of P0 and W4 samples along PC1 indicates that developmental stage is the dominant source of transcriptomic variation.

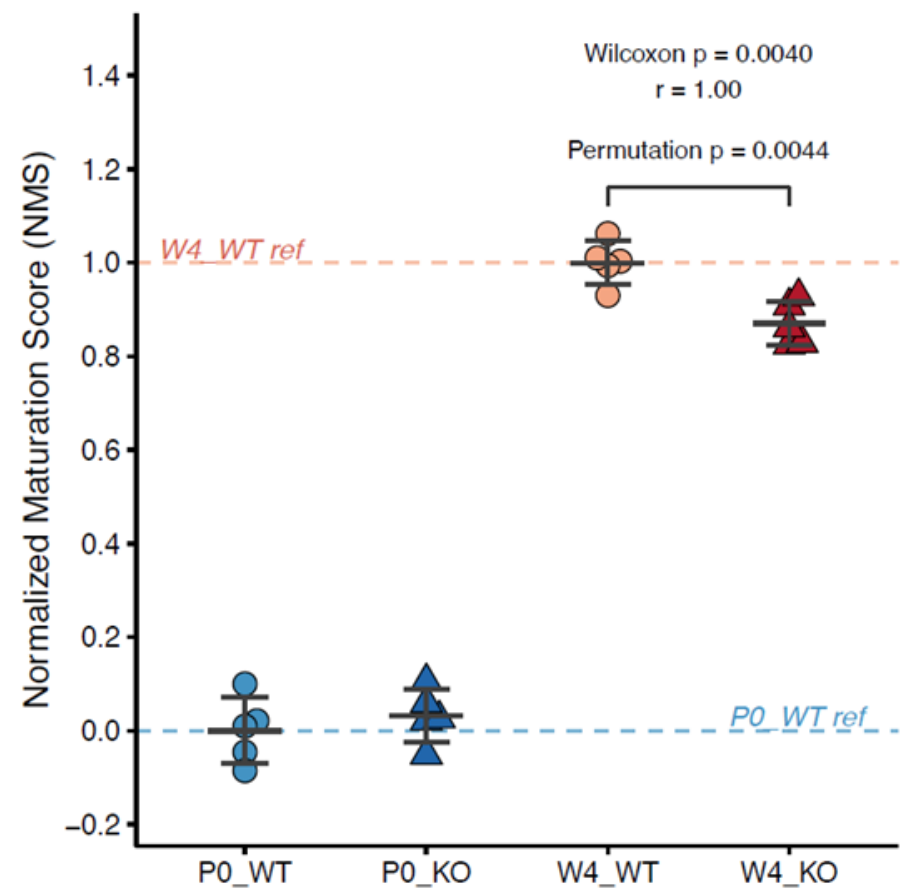

Fig S5. **Normalized Maturation Scores (NMS) of pituitaries from P0 and 4-week-old *Snord116* KO mice and wild type controls.** NMS of all four groups of samples, P0 and 4-week-old (W4) *Snord116* KO (KO) mice and wild type (WT) controls, were calculated based on WT maturation-associated genes, i.e., DEGs between P0_WT and W4_WT, and were linearly normalized to the P0_WT group mean and the W4_WT group mean, set to 0 and 1, respectively. W4_KO pituitaries reached only 87% of the W4_WT maturation level, which was statistically significant by one-sided Wilcoxon rank-sum test and permutation test (both *p*<0.01).

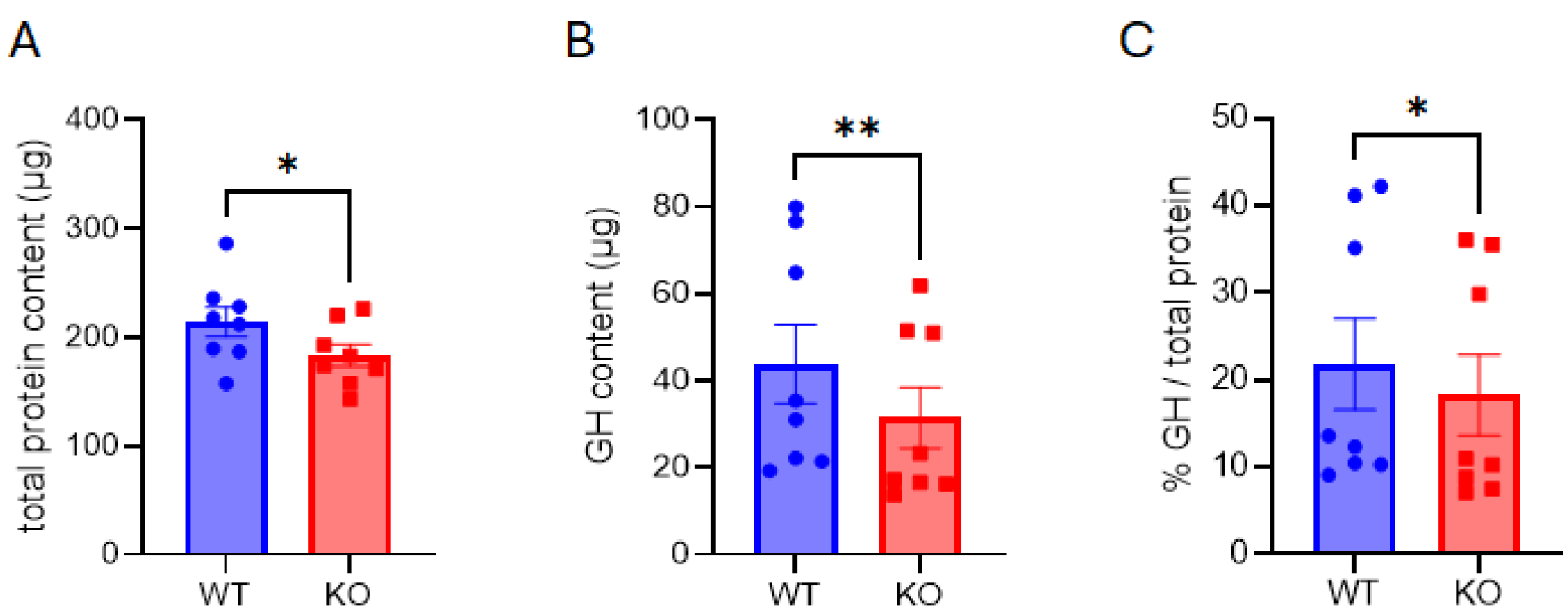

Fig S6. **Pituitary total protein and growth hormone (GH) contents are decreased in 5-week-old male *Snord116* KO mice relative to wild type controls**. **A-C**. Total protein content (**A**), GH content (**B**), and GH content normalized to total protein content (**C**) of the pituitaries of 5-week-old male *Snord116* KO mice and WT controls at the end of perifusion. Data are expressed as Mean ± SEM (n=8). * and ** denote p<0.05 and 0.01, respectively, KO vs WT by paired two-tailed t-test.

Supplemental Table 6: reagents and sources

**qPCR assays**		
**Gene name and assay type**	**Forward primer**	**Reverse primer**
mouse Snord116 SYBG qPCR	ATTCCCAGTCAAACATTCCTTGG	CAGTTCCGATGAGAGTGGCG
mouse Snrpn SYBG qPCR	ACGCTTGGTTCTGAGGAGTG	CCTTCGACGTTTGACCTGGA
mouse Ipw SYBG qPCR	AAGTGTCACCACAACACTGGAC	AAGCTGCTGGTAGAAGAAATGG
mouse Ppia SYBG qPCR	CCCACCGTGTTCTTCGACAT	CAAATCCTTTCTCTCCAGTGCTC
mouse Tbp SYBG qPCR	CAGCCTTCCACCTTATGCTCA	CTGTGGAGTAAGTCCTGTGCC
mouse 116HG Taqman		
PCR primers	AACTGTTGTGGTGTTGCTGACT	GGAGACTCAGTTGGACGACATAA
probe	AGGTTGCATTCCCTTTCCAGTATGGTCCATG-5' 6-FAM/ZEN/3' IB	
mouse Tbp Taqman		
PCR primers	AGCTCTGGAATTGTACCGCA	TGACTGCAGCAAATCGCTTG
probe	AG ACC ATT GCA CTT CGT GCA AGA AAT GCT G-5' HEX/ZEN/3' IBFQ	
**Genotyping and sex determination**		
**Gene name**		
mouse Snord116 WT, del alleles		
PA (Snordflox R7960)	TTTACGGTACATGACAGCACTCAAG	
PC (Snordflox R7958)	AATCCCCAACCTACTTCAAACAGTC	
PD (Snordflox R7959)	TGGATCTCTCCTTGCTTGTTTTCTC	
mouse Sry Taqman		
primers	GTGTCACAGAGGAGTGGCAT	GGAGTACAGGTGTGCAGCTC
probe	TGCAGTGGGACAGGAACCCACATGCC (5' 6-FAM/ZEN/3' IB)	
mouse Chr6 Taqman		
primers	TTCCCTCGTGATCTGCAACT	CACCTGTTCAATTCCC-CTGC
probe	TCTTCTGGGCAGGCTTAAAGGCTAACC T- (5' HEX/ZEN/3' IBFQ)	
**ELISA kit**		
**Protein name**	**Company**	**Cat No.**
mouse GH	Millipore-Sigma	EZRMGH
